# Veratrum Viride

**Published:** 1858-09

**Authors:** 


### VERATRUM VIRIDE.

We have received some time ago, and neglected to notice, a pamphlet upon this article, which has been attracting so much attention for a considerable time, and in reference to which there have been such widely discordant opinions, but which seems to be finally establishing for itself a most extraordinary reputation.

Until recently we have rather eschewed the article, our experience with it not having been sufficient to enable us to form any very decided opinion, but from some considerable use of it lately, we are enabled to state that it is a remedy of very great power for good, and nothing like so dangerous as we had been led to believe.

It is not, however, our purpose to enlarge upon the virtues of this agent at present, the principle object we have in view is, to express our regret, that Dr. Norwood should have so nearly destroyed the obligation imposed upon the Profession, in the *revival* of this remedy, by the *manner* in which it has been brought to their notice; and while we hope it has been, as stated by himself, his object to be useful to the profession and to benefit suffering humanity, we cannot help concluding, or at least fearing, that there has been some other object, when we find in his pamphlet upon this article, such remarks as, "If we had not already occupied so much time and space, we would give a *" *remedy for rheumatism and gout*, and for *puerperal convulsions*; also a *cure for scald-head*. What they are, and how they are used, shall appear in our next, when we prepare another *lot of Tincture* the next Spring or Fall." And again, "we can assure those who may use *our tinctnre*, that they can rely on its performing all we claim for it," &c.

The manner of introducing and advertising the article, we regard as irregular, and if we must say it, decidedly quackish. Why Dr. Norwood should be able to get a better drug from which the tincture is prepared, or why others cannot understand and execute the process of preparation, we are at a loss to understand. If making a tour through the United States, (as it is stated in one of the testimonials published in his circular, it was his intention to do,) "to *locate agencies* for the *sale* and more extensive dissemination of his invaluable discovery in the qualities and virtues of the Veratrum Viride," be entirely consistent with what is expected from the true man of science, and the gentlemanly and lofty bearing of the high-toned and philanthropic

* Italics are ours.

physician, then we must confess that our study of the ethics of the profession has been in vain.

Though it is true his formula for the preparation of Tincture of Veratrum, has been published, yet in the last edition of his pamphlet, while directions are given for using "*our tincture*," no place has been found for the process by which it is to be obtained, a very remarkable omission, it strikes us, if Dr. Norwood's tincture be so far superior to that to be obtained from other sources, and his object is, (as he professes,) "to be useful to his brethren in the profession, and to benefit suffering humanity."

For the sake of science, and that "suffering humanity," let us not degenerate into mere "*Tincture*" *makers*, or, *Hucksterers of physic*, and let every advance in Medical knowledge, at least, be as free as the wind.

